# Engaging Youth in Citizen-Led Advocacy and Accountability for Adolescent Sexual and Reproductive Health

**DOI:** 10.24248/eahrj.v3i2.603

**Published:** 2019-11-29

**Authors:** Sylvia Ayieko, Angela Nguku

**Affiliations:** a White Ribbon Alliance Kenya

## Abstract

The White Ribbon Alliance Kenya is an autonomous, locally registered nongovernmental organisation in Kenya with a vision to ensure that all women and girls realise their right to quality health and well-being. It is among 14 other national alliances affiliated with the White Ribbon Alliance Global Secretariat, based in Washington, DC. White Ribbon Alliance Kenya recognises that even though the Kenyan government has policies in place to improve adolescent sexual and reproductive health outcomes, proper implementation and commitment is still needed. Our advocacy and citizen-led accountability efforts are working towards educating youth on their sexual and reproductive health rights, obligations, and entitlements, and promoting public awareness and citizen engagement. The goal is to empower adolescents to demand high-quality sexual and reproductive health services through appropriate policy communication and advocacy channels.

## INTRODUCTION

The population in Kenya is estimated to be 43 million, with approximately 1 in 4 people classified as adolescents aged 10 to 19 years.^1,2^ Because they are more likely to engage in unprotected sexual intercourse and have limited perceptions of the associated risks, adolescents have a higher likelihood of contracting sexually transmitted infections (STIs), including HIV; adolescents are also at higher risk of pregnancy-related complications.^3^

Notably, 1 in every 5 adolescent girls aged 15 to 19 years is already a mother or is pregnant with her first child,^1^ and 1 in every 10 girls dies as result of a pregnancy-related complications.^4^ A recent study further revealed that adolescent girls accounted for 17% of all those seeking postabortion services and 45 % of all severe abortion-related complications in Kenyan hospitals.^5^ This high need for abortion services and the associated high abortion complication rate may be attributed to barriers in accessing family planning methods. In this regard, the unmet need for contraception among adolescents ages 15 to 19 stands at 23%,^1^ which is higher than all other age groups.

Unfortunately, adolescents often lack comprehensive knowledge about sexual and reproductive health, including available services; this increases the risk of morbidity and mortality. Understanding adolescent sexual and reproductive health needs and rights is therefore central to improving access, utilisation, and quality of services offered to this vulnerable group.

## CITIZEN-LED ADVOCACY AND ACCOUNTABILITY

Citizen-led advocacy and accountability emphasises the importance of a functional and transparent feedback mechanism as a driver of access to information that enables citizens to know and demand their rights and increase participation in decision-making processes, particularly regarding health. The White Ribbon Alliance Kenya has harnessed the passion, expertise, and power of citizens to accelerate progress for reproductive, maternal, and newborn health and rights. The organisation is always seeking to develop innovative, new, or improved approaches to increasing citizen demand and influencing change. Adolescent and youth health initiatives are most effective when they include young people as advocates for their own health. Without the direct involvement of young people, well-meaning efforts risk missing their intended mark. Why is this the case? First, youth are deeply affected by sexual and reproductive health outcomes that have long-term physical, emotional, psychological, social, and financial repercussions. Second, because of their inherent enthusiasm and energy, young people have the potential to maximise change and impact their contemporaries and society at large through citizen-led advocacy and accountability.

According to the World Health Organization, the Sustainable Development Goals (SDGs) will not be realised without investment in adolescent health and well-being.^6^ While the SDGs are a universal call to action to end poverty, protect the planet, and ensure that all people enjoy peace, prosperity, health, and well-being, recent gains have started to plateau in some countries and even regress in others. To achieve any new goals, including the SDGs, new approaches are needed.

In this regard, the White Ribbon Alliance Kenya is involved in elevating the voices of the youth by engaging them as effective advocates for improving adolescent sexual and reproductive health outcomes. The White Ribbon Alliance Kenya is an autonomous, locally registered nongovernmental organisation in Kenya with a vision to ensure that *all women and girls realise their right to quality health and well-being*. The organisation is among 14 other national alliances affiliated with the White Ribbon Alliance-Global based in Washington, DC. By involving youth in their communities, the White Ribbon Alliance Kenya recognises that advocacy galvanises ownership, engagement, fand uture involvement thereby yielding sustainable changes.

## STRATEGIES OF ENGAGEMENT

Historically, adolescent sexual and reproductive health programmes have focused on youth without letting them express what they want. The White Ribbon Alliance Kenya, in partnership with other civil society groups, supports Kenya's Ministry of Health in a phased approach to promoting youth-led advocacy by equipping youth and adolescents to become effective advocates for their own sexual and reproductive health. This entails educating and empowering youth about their sexual and reproductive health-related rights and entitlements. Whereas adolescent rights to health and development are enshrined in the Convention on the Rights of the Child,^7^ Article 43, 1(a) of the Kenyan Constitution,^8^ and other relevant human rights treaties, many young people are not aware of these rights and entitlements and thus cannot demand them. To date—and focusing on the most marginalised youth in our society—the White Ribbon Alliance Kenya has provided rights-based sexual and reproductive health education to over 40 males and females, aged 18 to 24 years, from selected informal settlements in Nairobi, including Kariobangi, Kibera, and Mukuru kwa Reuben.

The White Ribbon Alliance Kenya has also trained 23 young people on citizen journalism, with a focus on advocacy and accountability for reproductive, maternal, and adolescent health and rights. The training emphasised quality, equity, and dignity to reduce the appallingly high numbers of teenage pregnancies and maternal deaths among adolescents. The citizen journalists, including males and females aged 18 to 23 years, used their newly acquired skills to document, monitor, and track progress and gaps related to adolescent sexual and reproductive health, such as inadequate youth-friendly services as well as maternal and newborn health services in their subcounties. In collaboration with local media houses, they disseminated short documentaries about teenage pregnancy in their communities, with some sharing their communities’ experiences of rights abuses and violations. They also devised solutions to the problems, while creating a series of media posts on Twitter and Facebook, which have been shared locally and internationally.^9^

One of the most profound successes has been in gathering data for the *What Women Want Campaign: Demands for Quality Healthcare from Women and Girls*. This is a global campaign that seeks responses from women and girls about their desires related to quality maternal and reproductive health care. In Kenya, youth advocates in Nairobi, trained on citizen journalism, have served as mobilisers in conducting targeted outreach to ensure that the voices of marginalised and vulnerable populations—especially adolescent girls and young women in informal settlements—were captured in this campaign. The top 5 responses from adolescent girls and young women regarding what they want include: (1) respectful and dignified care; (2) water, sanitation, and hygiene; (3) increased numbers of competent and better-supported midwives, nurses, and doctors; (4) medications and supplies; and (5) menstrual health.^9^

The White Ribbon Alliance Kenya, with the support of the Population Reference Bureau, also brought together youth mentors from several subcounties in Nairobi to train them on policy advocacy and communication. The training took place in Nairobi County in July 2018 and involved 25 male and female youth mentors aged 18 to 24 years. These were selected from the 3 informal settlements based on their involvement in youth mentorship and reproductive health as well as in the citizen journalism training provided by the White Ribbon Alliance Kenya. The youth mentors acquired competencies regarding how to effectively communicate and advocate for proper implementation of sexual and reproductive health policies in Kenya, such as the Return to School Policy and the National Adolescent Sexual Reproductive Health Policy to leverage for improved adolescent sexual and reproductive health. At the end of the training, they proposed solutions, such as reaching out to school-going children and other youth groups in their communities. Subsequently, some of the youth mentors have been invited to speak to teenage mothers on adolscent and youth sexual and reproductive health issues in various schools and churches, while others have engaged in community dialogues for youth to address specific adolescent sexual and reproductive health issues, contextual to their communities, STIs/HIV, and high-quality reproductive health services in health facilities.

We note that these strategies and innovative approaches have been successfully used to engage youth in adolescent and youth sexual and reproductive health advocacy.

## CONCLUSION

Citizen-led advocacy among youth is a small but fundamental step towards improving adolescent sexual and reproductive health outcomes in Kenya. Solving the high burden of teenage pregnancy, maternal mortality, and STI/HIV rates among adolescents requires a multifaceted approach and the dedication and commitment of all stakeholders, including the adolescents themselves. The government, through the Ministry of Health, should reinforce sexual and reproductive health policies targeting youth and ensure multisectoral collaboration, including with the ministries of education, gender, finance, and planning. Development and funding partners, faith-based organisations, civil societies, the media, and the private sector are further urged to partner with organisations promoting citizen-led advocacy for adolescent sexual and reproductive health. The White Ribbon Alliance Kenya continues to advocate for increased investment in social accountability initiatives targeting adolescents and young people, taking cognisance of the clarion call “Nothing about us without us”.
